# Development and validation of a new multiplex for upgrading Y-STRs population databases from 12 to 23 markers and its forensic casework application

**DOI:** 10.1038/s41598-022-25785-z

**Published:** 2022-12-16

**Authors:** Belén Navarro-López, Miriam Baeta, Eva Granizo-Rodríguez, O. Moreno-López, Tamara Kleinbielen, Joana Francesca Ferragut, Antònia Picornell, Marian M. de Pancorbo

**Affiliations:** 1 BIOMICs Research Group, Lascaray Research Center, University of the Basque Country UPV/EHU, Vitoria-Gasteiz, Spain; 2 Bioaraba Health Research Institute, 01009 Vitoria-Gasteiz, Araba Spain; 3 Departament de Biologia Institut Universitari d’Investigació en Ciències de la Salut (IUNICS) i Laboratori de Genètica, Universitat de les Illes Balears, Carretera de Valldemossa, km 7.5, 07122 Palma de Mallorca, Illes Balears Spain

**Keywords:** Population genetics, Genetic markers

## Abstract

Y chromosomal short tandem repeats (Y-STRs) are used in forensic investigations as a useful complementary tool to autosomal markers. The ongoing development of new kits with an increasing number of markers makes it necessary to update populations typed in the Y-STR Haplotype Reference Database to reach at least 23 Y-STRs. A novel Y-STR multiplex panel was developed to offer a cost-efficient alternative to update Y-STR haplotypes from 12 to 23 loci. This panel includes the eleven markers, DYS448, DYS456, DYS458, DYS635, Y-GATA H4, DYS576, DYS481, DYS549, DYS533, DYS570 and DYS643, as well as DYS385a/b for traceability purpose. Developmental validation of this panel was conducted following the recommendations of the Scientific Working Group on DNA Analysis Methods (SWGDAM), showing high sensitivity, tolerance to common inhibitors as well as high species specificity. It was efficient for degraded DNA samples and for detection of male mixtures. When applying it for extending the current data of the Ibiza population, both the discrimination capacity and the haplotype diversity increased from 0.5952 to 0.9048 and from 0.9808 to 0.9977, respectively. Together, the study demonstrates the suitability of this panel in forensic casework.

## Introduction

Y chromosome polymorphisms have become a valuable tool in forensic, anthropological, evolutionary, and genealogical studies^1,2^. Due to its paternal inheritance, they elude recombination to a great extent, being able to discriminate between male lineages, preserving a simple record of their history and being useful tools for understanding the geographical distribution of current populations^3^. Y-STRs, with mutation rates in the order of 1 × 10^–3^ per locus per generation, have strong power of discrimination among paternal unrelated lineages and play a key role in the field of forensic genetics^2,4–7^.

Although Y-STRs do not allow individualization as autosomal markers do, they may be useful in certain situations. For instance, for male donor analysis in male–female DNA mixtures, such as in cases of sexual assault, paternity testing with male offspring^8–10^ and disaster victims or missing person investigations, involving males^11^. Furthermore, Y-STR information has proven to be helpful in rapidly determining the number of male contributors in mixtures. Being hemizygous, Y-STR profiles have mostly one allele in their loci, so the presence of multiple alleles at single-copy loci is a clear clue of the number of male contributors^10,12,13^. Y-STR analysis can also aid in reconstructing paternal relationship and even sometimes be used to infer the geographic region of paternal ancestry of a male DNA donor, useful in the case of missing persons^1,9,10^.

Over the past decades, thousands of Y-STRs have been identified and several commercially available Y-STR kits have been developed^14^. Currently, most studies related to the analysis of Y-STRs aim to search for new markers to develop multiplex panels that offer greater discriminatory power, as it has been observed that a low number of Y-STRs may be insufficient for differentiating male lineages^15^. This is the reason why more markers are needed than those included initially in the 9 Y-STRs of the Minimal Haplotype, the 12 Y-STRs included in the PPY (PowerPlex^®^Y, Promega Corporation, Madison, WI, USA), or the 17 Y-STRs of the Yfiler® kit (Yfiler, Life Technologies, Foster, City, CA)^1^. Indeed, the development of new kits with a larger set of markers has been shown to provide higher genetic resolution by increasing both haplotype diversity and discriminatory power compared to other panels in numerous populations^16–21^.

Despite the foregoing, where more markers analysed usually means greater discriminatory power, to date numerous Y-STR population data available in the Y-STR Haplotype Reference Database (http://www.yhrd.org) are still based on the 12 Y-STRs of the PPY. In fact, currently the YHRD (Release R67) still presents 1398 population samples including 9 Y-STRs (Minimal Haplotype), 1177 with 12 Y-STRs (PPY) and 1089 containing 17 Y-STRs (Yfiler). However, only 402 samples report the 23 Y-STRs of the PPY23 (PowerPlex^®^ Y23 System, Promega Corporation, WI, USA) and 314 the 27 Y-STRs of the YfilerPlus^22^. The ideal scenario would be for the same samples to be regenotyped as new markers appear. This would allow the databases to be updated continuously, which would be of great advantage in the forensic field. However, re-analysing all populations with the new Y-STR kits that are becoming commercially available is a labour-intensive, time-consuming and expensive task.

**Figure 1:**
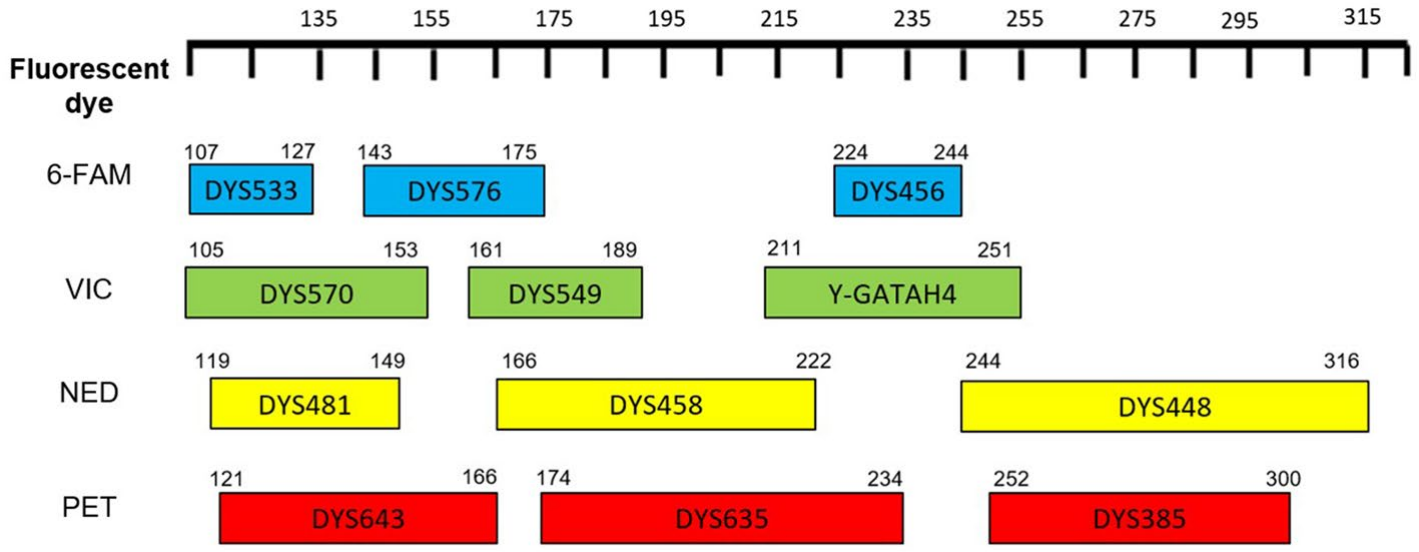
Design of the 11 plus DYS385a/b Y-STR multiplex panel developed in the present study. The boxes represent the expected fragment sizes for each locus and the color of the fluorescent dye with which forward primers were labeled. The scale at the top represents the size (bp) and the fluorescent dye for the corresponding loci is shown on the left.

Here, we present the development and validation of a novel 11 plus DYS385a/b Y-STR multiplex panel, an extension of the one previously developed by Nuñez et al. (2017)^15^. The extended panel includes the eleven markers, DYS448, DYS456, DYS458, DYS635, Y-GATA H4, DYS576, DYS481, DYS549, DYS533, DYS570 and DYS643, which are contained in the PPY23 but absent in the PPY, as well as DYS385a/b for traceability purposes (Fig. 1). All loci here included and their mutation rates have been previously studied^1,23,24^. DYS385a/b has been selected as an overlapping marker since it is a multicopy Y-STR and reaches the highest genetic diversity among the markers present in PPY23^17^. The number of markers has been extended to 23 because, as previously mentioned, among the kits with a high number of markers, PPY23 is the one with the largest proportion of samples analysed. The aim of this new panel is offer a more cost-effective option to update populations that are currently typed with PPY. In addition, this would allow us to perform comparative analyses of the updated populations with a greater number of individuals with a genetic profile of 23 Y-STRs. The primer design was based on a miniSTR strategy in order to increase the performance success of this multiplex in degraded DNA. The developmental validation was performed following the guidelines of the SWGDAM^25^. The new multiplex was applied in a population that was previously analyzed for the 12 Y-STRs of PPY with the aim to increase the current genetic data as well as to evaluate the capability of the multiplex.

## Material and methods

### Selection of markers and primer design

The new multiplex panel includes the eleven markers DYS448, DYS456, DYS458, DYS635, Y-GATA H4, DYS576, DYS481, DYS549, DYS533, DYS570 and DYS643, which are contained in the PPY23 but absent in the PPY. Additionally, the loci DYS385a/b was included in the multiplex reaction as a traceability marker, as it is present in the aforementioned commercial kits.

Since the 11 plus DYS385a/b Y-STR multiplex panel is an extension of the earlier one developed by Nuñez et al. (2017), for the eight markers that were already contained in that panel (DYS456, DYS576, DYS481, DYS549, DYS533, DYS570, DYS643 and DYS385a/b) the same primers described were used^15^. For the remaining four Y-STR loci analyzed (DYS448, DYS458, DYS635 and Y-GATA H4), the primer design was performed using PerlPrimer v1.1.21^26^. Potential primer cross-reactions were examined via this software, and the Y-chromosome specifity was evaluated using BLASTN aligment tool (https://blast.ncbi.nlm.nih.gov/Blast.cgi). Forward primers were labeled with a fluorescent dye at the 5′ end (Supplementary Table S1).

### Singleplex PCR reaction

Each primer pair was initially tested in a singleplex PCR reaction using the 2800 M control DNA (Promega Corporation, Madison, WI). The singleplex reaction consisted of 12.5 μl of QIAGEN Multiplex PCR kit (Qiagen, Valencia, CA), 1 μl of primer (forward and reverse with an initial concentration of 10 μM), 1 ng of genomic DNA and 10.5 μl of Milli-Q water for a final reaction volume of 25 μl. PCR was performed in a GeneAmp 9800 (AB/LT/TFS) under the following cycle conditions: initial denaturation at 95ºC for 15 min was followed by 30 cycles of 94 °C for 30 s, 65 °C for 90 s, and 72 °C for 90 s, and a final extension at 72 °C for 10 min. Evaluation of amplification performance was conducted by electrophoresis on 1.50% agarose gels and visualized with GelRed (3.00% μL/ml) (Biotium Inc.,Hayward, USA) and UV light (UVItec Cambridge, USA or UK). PCR products were purified using 2 μl EXOSAP (Takara Bio Inc., Japan) to 5 μl of PCR product and sequencing was performed to confirm the specific amplification of each Y-STR. Sequencing was performed using the BigDye^®^ TerminatorTM v1.1 Cycle Sequencing Kit (AB/LT/TFS: Applied Biosystems™, Life Technologies, ThermoFisher Scientific, Waltham, MA, USA). Sequencing products were purified with BigDye^®^ XTerminatorTM Purification Kit (AB/LT/TFS) and capillary electrophoresis was done on a 3130 Genetic Analyzer (AB/LT/TFS). Sequencing results were analyzed with the Sequencing Analysis software v5.2 (AB/LT/TFS).

### Multiplex PCR reaction and capillary electrophoresis

The 12 Y-STR loci under study were typed using multiplex reactions containing 5 μl of QIAGEN Multiplex PCR kit (Qiagen, Valencia, CA), 0.8 μl of primermix (primer concentrations in the mix are described in Supplementary Table S1),1 ng of genomic DNA and Milli-Q water for a final reaction volumen of 10 μl. PCR was performed in a GeneAmp 9800 (AB/LT/TFS) using the same foregoing cycle conditions. PCR products separation and detection were performed by capillary electrophoresis with an ABI3130 Genetic Analyzer (AB/LT/TFS) using the GeneScan 500 LIZ (AB/LT/TFS) as an internal size standard and fragment lengths were assessed with GeneMapper v4.0 (AB/LT/TFS). The analytical threshold was set at 50 RFUs for peak height minimum. Allelic nomenclature follows the recommendations of the International Society for Forensic Genetics (ISFG) (https://www.isfg.org).

The allelic ladder for this multiplex was constructed (Supplementary Fig. S1). For each Y-STR locus, DNA samples showing different allelic variations were simultaneously amplified using the corresponding primer pair. Amplification product for each allele was mixed at appropriate ratios to produce the multiplex allelic ladder.

### Concordance study

To compare allele designation concordance of the new assay, a set of 100 male DNA samples from the resident population living in the Basque Country previously analyzed with PPY23 in our laboratory, was examined with the novel multiplex panel. These two assays have in common the 12 Y-STR markers included in the new multiplex.

### Sensivity and stability studies

In order to evaluate the minimum amount of DNA required to obtain a complete Y-STR profile, 2800 M DNA control (Promega, Madison, WI) was used for amplification in triplicates in ascending quantities: 25 pg/μl, 50 pg/μl, 100 pg/μl, 200 pg/μl, 400 pg/μl, 1 ng/μl, 1.6 ng/μl, and 10 ng/μl.

Stability studies were conducted by including different concentrations of two common inhibitors, haematin (Sigma-Aldrich Corporation, St. Louis, MO, USA) and humic acid (Sigma-Aldrich Corporation, St. Louis, MO, USA) to the amplification reaction mix containing 1 ng of 2800 M DNA. This study was performed in duplicate using ascending concentrations of the PCR inhibitors: 100 μM, 150 μM, 300 μM, 500 μM, 750 μM, 1000 μM, 1500 μM, 3000 μM, and 5000 μM of haematin and 25 ng/μl, 50 ng/μl, 100 ng/μl, 200 ng/μl, 250 ng/μl, 300 ng/μl, 500 ng/μl, 1000 ng/μl, 2000 ng/μl, and 3000 ng/μl of humic acid.

Artificially degraded DNA samples were prepared to evaluate stability of the Y-STR panel. 1 μg of a DNA in-house control sample was digested with 1 μL of DNAse Reaction Buffer, 0.5 μL of DNAse I and Milli-Q water for a final reaction volumen of 10 μl (DNase I, RNase-free, ThermoFisher Scientific, Waltham, MA, USA) for 15 min, 30 min, 1 h, 2 h, 4 h and 16 h at 37 °C. To inactivate DNAse I, 1 μL of 50 mM EDTA was added and then incubated at 65 °C for 10 min. These samples were amplified in duplicate.

### Species specifity

To analyze the species specificity, DNA samples from bull, goat, sheep, pig, cock, rabbit, dog, cat and mouse were tested. All samples were provided by the Bank of DNA of the BIOMICs Group at the University of the Basque Country (UPV/EHU). Of each animal species, 1 ng of DNA was used for each amplification reaction.

### Repeatability and reproducibility

The parameter of repeatability was evaluated by analysing whether the peaks of different replicas were always located in the position where the allele of the corresponding STRs should appear.

Reproducibility was checked by performing the entire analysis by three different operators and by running amplification PCRs on three different thermal cyclers: GeneAmp PCR System 9700 Gold (AB/LT/TFS), GeneAmp 9800 PCR System (AB/LT/TFS) and C1000 thermal cycler (Bio-Rad, Hercules, CA, USA). Afterwards, the electropherograms resulting from these diverse analyses were compared to test the reproducibility of the results.

### Mixture detection

The capacity of our panel to detect an admixed contribution was tested with a male:male and male:female mixtures. Male:male DNA mixtures were prepared using 2800 M DNA control and DNA in-house control sample with the following ratios of 19:1, 9:1, 3:1, 1:1, 1:3, 1:9 and 1:19. To perform the multiplex amplification reaction the total amount of mixed DNA input was mantained at 1 ng. Male:female DNA mixtures were established using a constant amount of 30 ng K562 DNA control (Promega) with a decreasing template of 2800 M DNA control (1 ng, 400 pg, 200 pg, 100 pg, 50 pg and 25 pg). Each mixture analysis was repeated for two times.

### Sizing accuracy and stutter calculation

Sizing accuracy was tested by evaluating the standard deviation of allele size observed after 20 injections of the allelic ladder (Supplementary Fig. S1) on a 3130 Genetic Analyzer (AB/LT/TFS).

Peaks that differed from the true allele by one repetition (± 0.5 bases) were considered stutter peaks. The percentage of observed stutter at each locus was examined by dividing the stutter peak height (in RFUs) by the corresponding allele peak height. To evaluate the effect of stutter peaks, a subset of 100 samples were randomly selected from the total collection analyzed in this study.

### Analysis of casework-type samples

To test the effectiveness of this panel with forensic-type samples, two samples from skeletal remains (bones) with degraded DNA and three casework samples from intercomparison exercises organized by the Spanish and Portuguese-Speaking Group (GHEP) of the International Society for Forensic Genetics (ISFG), were analyzed. Samples from skeletal remains (coded in this study as BADN1304 and BADN2049) belong to Spanish Civil War (1936–1939). These samples were selected as examples of forensic samples routinely analysed in our laboratory. The samples from GHEP were items M4 and M8 from exercise 2018 (coded in this study as M4-GHEP18 and M8-GHEP18) and item M4 from exercise 2020 (coded in this study as M4-GHEP20).

### Population study and forensic parameters

A sample of 84 unrelated males from Ibiza (Balearic Islands, Spain), previously genotyped with the 12 Y-STR PPY^27^ was combined with the new Y-STRs in order to assess the forensic parameters of the resulting 23 Y-STR haplotypes.

The study was approved by the General Directorate of Research + Development + Innovation of the Government of the Balearic Islands, Spain (Ref. AAEE procedure 12099/2003). All donors gave their informed consent before inclusion in the study, following the principles and ethical guidelines of the Declaration of Helsinki for the protection of human subjects. The individuals from this study also gave their informed consent for the samples to be used in the population-based follow-up studies. Samples were collected in 2003. According to current Spanish legislation, samples collected before 2007 can continue to be used for research purposes, for which they must be anonymized. Blood samples used in this work were collected before 2007 and anonymized in accordance with the Law 14/2007 on Biomedical Research. DNA was extracted by using QIAamp spin columns (Qiagen, Hilden, Germany) following the manufacturer’s recommendations.

Allele frequencies, single-marker genetic diversity (GD), haplotype diversity (HD), as well as different, unique and population specific haplotypes, were assessed using the software Arlequin v3.5.2.2^28^. For these calculations, the DYS385a/b alleles were treated as haplotypes. The global discrimination capacity (DC) was calculated by dividing the number of different haplotypes by the total number of individuals in the population sample.

### Ethic declarations

The study was approved by the General Directorate of Research + Development + Innovation of the Government of the Balearic Islands, Spain (Ref. AAEE procedure 12099/2003). All donors gave their informed consent before inclusion in the study, following the principles and ethical guidelines of the Declaration of Helsinki for the protection of human subjects. The individuals from this study also gave their informed consent for the samples to be used in the population-based follow-up studies. Samples were collected in 2003. According to current Spanish legislation, samples collected before 2007 can continue to be used for research purposes, for which they must be anonymized. Blood samples used in this work were collected before 2007 and anonymized in accordance with the Law 14/2007 on Biomedical Research.

## Results and discussion

### Primer set and PCR optimization

The 11 plus DYS385a/b Y-STR multiplex panel allows the analysis of the markers DYS448, DYS456, DYS458, DYS635, Y-GATA H4, DYS576, DYS481, DYS549, DYS533, DYS570, DYS643 and DYS385a/b. Primer design was carried out following a miniSTR approach by locating primers as close to the Y-STR repetition units as possible. The final 12 primer pairs generate PCR fragments 105 to 316 bp long (Fig. 1 and Supplementary Table S1). This design has the advantage of reducing the amplicon size for markers DYS533, DYS549, DYS481, DYS643 and Y-GATA H4 with regard to the PPY23 system^29^ (Supplementary Fig. S2).

### Concordance study

A total of 100 samples from the resident population living in the Basque Country were compared with haplotypes obtained in previous analyses performed in our laboratory employing the PPY23 kit. The results showed that the profiles of the same 12 Y-STR markers were completely identical.

### Sensivity and stability studies

Sensitivity was evaluated through the analysis of positive control 2800 M at different concentrations. The results indicated that the minimum quantity of DNA necessary to obtain complete genetic profiles, with peak heights above 50 RFUs, in triplicate reactions was 100 pg/μl. Lower amounts experienced allele dropouts of the larger amplicons (Supplementary Table S2 and Supplementary Figs. S3 and S5). However, given the results for 50 pg/μl and 25 pg/μl, by increasing certain conditions within the genotyping step, such as the amount of DNA or the injection time, complete profiles might be obtained. Taken together, these results suggest that 11 plus DYS385a/b Y-STR multiplex panel under study could be applied to forensic remains with high limitation in their DNA content.

Stability studies consisted of PCR reactions that included inhibitors such as haematin or humic acid. Complete genetic profiles were obtained with concentrations of ≤ 100 μM of haematin and ≤ 50 ng/μl of humic acid for all replicas (Supplementary Table S2 and Supplementary Figs. S4 and S5). Additionally, to determine the efficiency of amplification with degraded samples, artificially degraded DNA was prepared by digestion with DNAse I at different times. Complete genetic profiles were obtained with incubation times < 1 h for all replicas. After 1 h incubation, due to the shortage of target DNA, the results start to randomise. For instance, the 1 h incubation time resulted in 83.33% and 50.00% partial profiles, for each replica respectively. Another example occurs after 4 h incubation, where 50.00% and 16.67% of partial profiles were obtained for each replicate respectively (Supplementary Figs. S6 and S7). All stability test results are indicative of the robustness of the novel panel at moderate DNA degradation levels.

### Species specifity

Evidence collected at the crime scene may be exposed to non-human biological sources, such as those of animal origin, and cross-contamination with DNA from other species may occur. In this regard, it is important to assess the species specificity of the assay, ensuring that primers designed to amplify the Y-STRs in this 11 plus DYS385a/b Y-STR multiplex panel are human-specific and do not amplify animal DNA. Here, we have analysed some common livestock and domestic animal species, assuming that these might be the most frequently found at a crime scene.

The results showed the absence of DNA amplification results for all non-human samples tested (Supplementary Fig. S8). Therefore, our panel produces no cross-reaction in the tested animal samples, which shows its specificity in terms of species that are common in the human habitat.

### Repeatability and reproducibility

Analysis of different replicas proved the repeatability of the panel. No profile experienced a change of allele in any of the replicates, and the position in which the peaks corresponding to each marker appeared did not suffer displacements in the different electropherograms. Since samples are analysed in different capillaries and different runs, the background noise may differ between replicas, resulting in electropherograms with slightly different RFUs.

Moreover, in order to check the reproducibility of the technique when using different thermocyclers, the procedure was performed in a GeneAmp PCR System 9700 (AB/LT/TFS), in a GeneAmp PCR System 9800 (AB/LT/TFS) and in a C1000 Thermal Cycler (Bio-Rad, Hercules, CA, USA). No differences were observed in the results, demonstrating the reproducibility of the technique when different thermocyclers were used.

Additionally, no further differences were detected when the technique was performed by three different operators. In all cases, identical electropherograms were obtained, resulting in complete profiles of 12 Y-STRs.

### Mixture detection

Mixtures of multiple male samples or female and male are frequently encountered in forensic crime scenes. Here, the ability of the panel to reveal the presence of mixed DNA samples was tested by two types of mixtures containing male:male and male:female.

**Figure 2:**
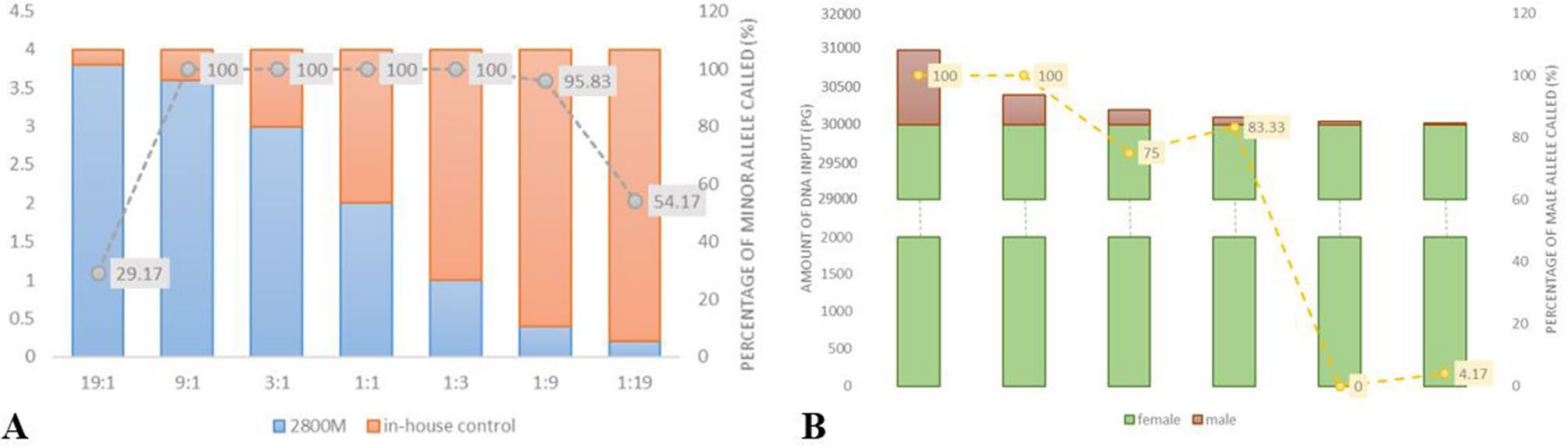
Percentages of allele recovery for the minor contributor with ratios varying from 19:1 to 1:19 in male:male mixture study (**A**). Percentages of allele recovery in male:female mixtures decreasing the male DNA from 1 ng to 25 pg and keeping constant the female DNA to 30 ng (**B**).

For male:male DNA mixtures, as the ratios increased, the proportion of minor alleles that could be identified decreased. The minor component at 3:1 and 1:3 ratios was completely identified. At 9:1 the minor component resulted also on complete profiles. However, the minor component at 1:9 resulted in a 95.83% partial profile on average. At ratios of 19:1 and 1:19, the minor component resulted in 29.17% and 54.17% partial profiles on average, respectively (Fig. 2a, Supplementary Table S3 and Supplementary Fig. S9a).

For male:female DNA mixtures, 11 plus DYS385a/b Y-STR multiplex panel was tested in the presence of large amounts of female DNA. The results showed that complete profiles were obtained when the male DNA amount was varied from 1 ng to 400 pg. When the male template DNA amount was reduced to 200 pg, 100 pg, 50 pg and 25 pg the average loci detection rates declined due to allele dropout (Fig. 2b, Supplementary Table S3 and Supplementary Fig. S9b). A higher percentage of alleles recovered in 100 pg compared to 200 pg, as well as in 25 pg compared to 50 pg, is due to the fact that primer annealing during PCR is a random process. In cases where there is a small amount of DNA, this randomness can lead to such results. Furthermore, obtaining complete profiles with a higher amount of DNA than in the sensitivity test is explained by the fact that sensitivity might be affected in mixtures. When the target sequence represents only a small fraction of the total DNA, as in this case where 30 ng of female DNA control is present, the annealing frequency between the primers and the target sites in the first cycles can be reduced, leading to a decrease in sensitivity^30^.

Identifying the number of contributors in the mixtures and defining the minor and major contributor genotyping, as well as obtaining the Y-STR profile of male contributor in sexual assault cases are some of the challenges of forensic investigation. The 12Y-STR multiplex panel has proven to overcome these difficulties successfully.

### Sizing accuracy and stutter calculation

To test the sizing accuracy of amplified alleles, size differences between the alleles from 20 injections of the allelic ladder on a 3130 Genetic Analyzer (AB/LT/TFS) were compared. The standard deviation in the size values was calculated for each allele in each locus (Supplementary Table S4). It ranged from 0.0355 (DYS481) to 0.1856 (DYS385ab), suggesting high accuracy of the detection system and demonstrating that the size precision level is sufficient for sizing and discrimination of microvariants and off-ladder peaks.

Stutter peaks are common artifacts observed during the PCR amplification process. Since these artifacts can complicate interpretation, it is important to evaluate the expected percentage of stuttering at each locus to avoid incorrect allele sizing^31^. Stutter parameters are shown in Supplementary Table S5. DYS643 was the locus with the lowest stutter ratio percentage (4.72%) while the higher average percentage was showed by DYS481 (21.32%). Except for the loci of DYS456 (17.26%) and DYS481 (21.32%), the mean stutter ratios of other loci were lower than 15%.

### Analysis of casework-type samples

The effectiveness of this panel for analyzing forensic-type samples was tested over two skeletal remains with degraded DNA, as well as over three items from intercomparison exercises organized by the GHEP-ISFG. The analysis of skeletal samples from Spanish Civil War (1936–1939), BADN1304 and BADN2049 resulted on complete profiles (12 Y-STRs). These results were compared with the ones obtained with the PPY23, and in both cases the same Y-STR profiles were obtained. The analysis of samples M4-GHEP18 and M8-GHEP18 resulted in complete profiles. These results were checked with the consensus results obtained in the intercomparison exercises of GHEP-ISFG, confirming the Y-STR profile of these samples and reaffirming the reliability of this panel. M4-GHEP20 is a mixture of blood and semen from two males being possible to observe both complete profiles in its analysis. Overall, these results show that the electropherograms obtained with this panel can be informative enough for forensic-type samples, even if they contained degraded DNA.

### Population study and forensic parameters

The 11 plus DYS385a/b Y-STR multiplex panel was applied to expand the current data available for the population of Ibiza, previously analyzed with the PPY. Forensic parameters were compared for the 12 loci previously analyzed (PPY) and the resulting 17 Y-STRs (contained in Y-Filer) and 23 Y-STRs (contained in PPY23), both using PPY data extended with the novel 11 plus DYS385a/b Y-STR multiplex panel.

#### Allelic frequencies and haplotypes

Allele frequencies and gene diversity (GD) of the 23 Y-STRs (12 Y-STRs previously analyzed and the new 11 Y-STRs here genotyped) for Ibiza are summarized in the Supplementary Table S6. Supplementary Table S7 provides the 23-loci haplotypes of the 84 individuals from this population.

#### Forensic and population genetic parameters

At the single-locus level, DYS456, DYS458 and DYS576 loci were the most discriminative of the eleven incorporated Y-STRs included in the multiplex, generating the highest GD values (0.8181 for DYS576, 0.7533 for DYS458 and 0.7372 for DYS456) (Supplementary Table S6). The lowest GD value in this case was 0.4079 for DYS533. These results are maintained if DYS385a/b loci is included in the computation.

At the haplotype level, the forensic and population genetics parameters of the Ibiza population based on 12-loci contained in PPY (12 loci previously analyzed with PPY), 17-loci contained in Y-Filer (12 loci previously analyzed with PPY and new 5 loci here analyzed with the 11 plus DYS385a/b Y-STR multiplex panel) and 23-loci contained in PPY23 (12 loci previously analyzed with PPY and new 11 loci here analyzed with the 11 plus DYS385a/b Y-STR multiplex panel) haplotypes are compiled in Table 1. As expected the values for number of haplotypes, unique haplotypes, haplotype diversity and discrimination capacity augmented as the number of loci increased. When 23-loci haplotypes were considered, Ibiza exhibit high levels of haplotype diversity, with value of 0.9977, and the discrimination value was 0.9048.

**Table 1:** Diversity parameters obtained for the population analyzed considering 12, 17 and 23 loci.

Ibiza	12 loci (previously analysed with PPY)	17 loci (12 loci previously analysed with PPY and new 5 loci here analysed with the 11 plus DYS385a/b Y-STR multiplex panel)	23 loci (12 loci previously analysed with PPY and new 11 loci here analysed with the 11 plus DYS385a/b Y-STR multiplex panel)
N	84	84	84
Different haplotypes	50	64	76
Unique haplotypes	32	49	68
HD	0.9808 ± 0.0056	0.9923 ± 0.0034	0.9977 ± 0.0022
DC	0.5952	0.7619	0.9048

*N* sample size, *HD* haplotype diversity, *DC* discriminatory capacity.

## Conclusion

In the present study, a novel 11 plus DYS385a/b Y-STR multiplex panel has been developed and validated to expand to 23-loci Y-STR haplotypes population samples already analyzed with the 12-Y-STRs (PPY). Indeed, the application of this new panel has allowed to update the current Y-STR database of the Ibiza population previously based on 12 loci genotyped by using the PPY, increasing the resulting haplotype diversity and discriminatory capacity, and consequently providing evidence of the suitability of these Y-STR markers for forensic purposes. Furthermore, the validation studies have also shown that the new panel is valuable for forensic applications as it overcomes the typical difficulties found at a crime scene. In conclusion, the new panel here described represents an efficient and affordable alternative to expand to 23 Y-STRs the studied markers in numerous populations that nowadays are analyzed only with the PPY, being of great interest in population genetics and forensic use.

## Supplementary Information

Supplementary Figures.

Supplementary Tables.

## Data Availability

The datasets generated and analysed during the current study are deposited in the YHRD repository, (accession number: YA003484), https://yhrd.org/YA003484.
