# Supplementary material for: Development and validation of a new multiplex for upgrading Y-STRs population databases from 12 to 23 markers and its forensic casework application
Source: Sci Rep. 2022 Dec 16;12:21734. doi: 10.1038/s41598-022-25785-z (PMC9758231; doi:10.1038/s41598-022-25785-z)
Supplement: Supplementary file 1 — Supplementary Figures. [file 41598_2022_25785_MOESM1_ESM.docx]

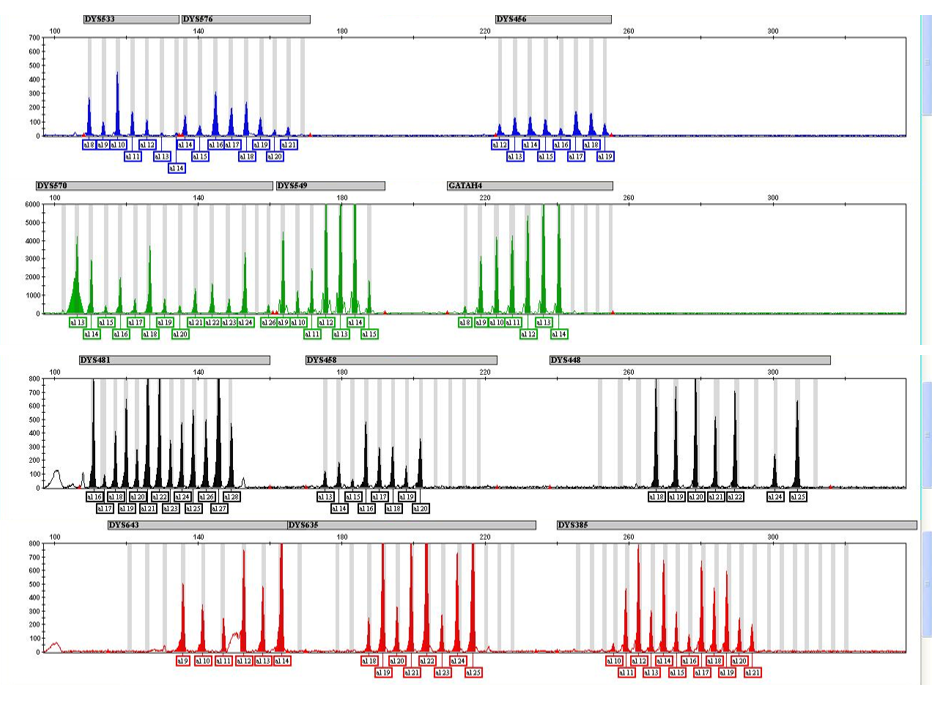


**Supplementary Figure S1.** Electropherogram of the allelic ladder for the 11 plus DYS385a/b Y-STR multiplex panel.


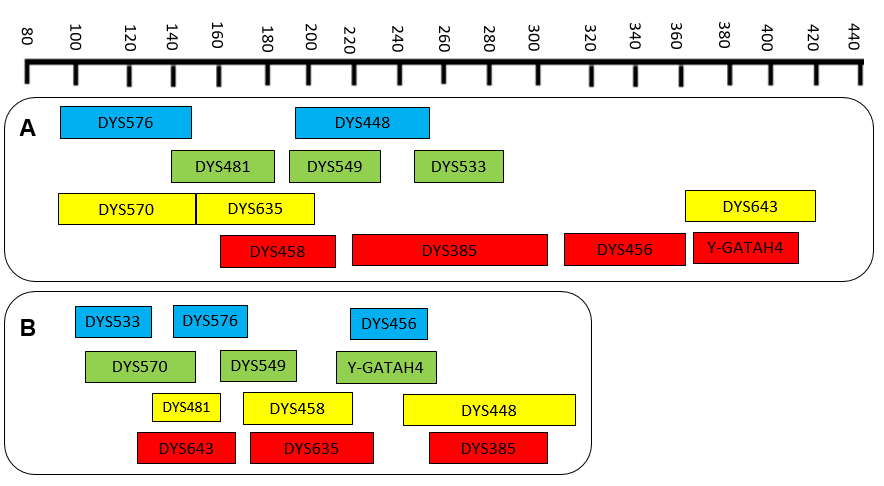


**Supplementary Figure S2.** Diagram of the fragment size for the DYS533, DYS576, DYS456, DYS570, DYS549, Y-GATAH4, DYS481, DYS458, DYS448, DYS643, DYS635 and DYS385 markers included in the PPY23 (A) and the panel developed in the present study (B).


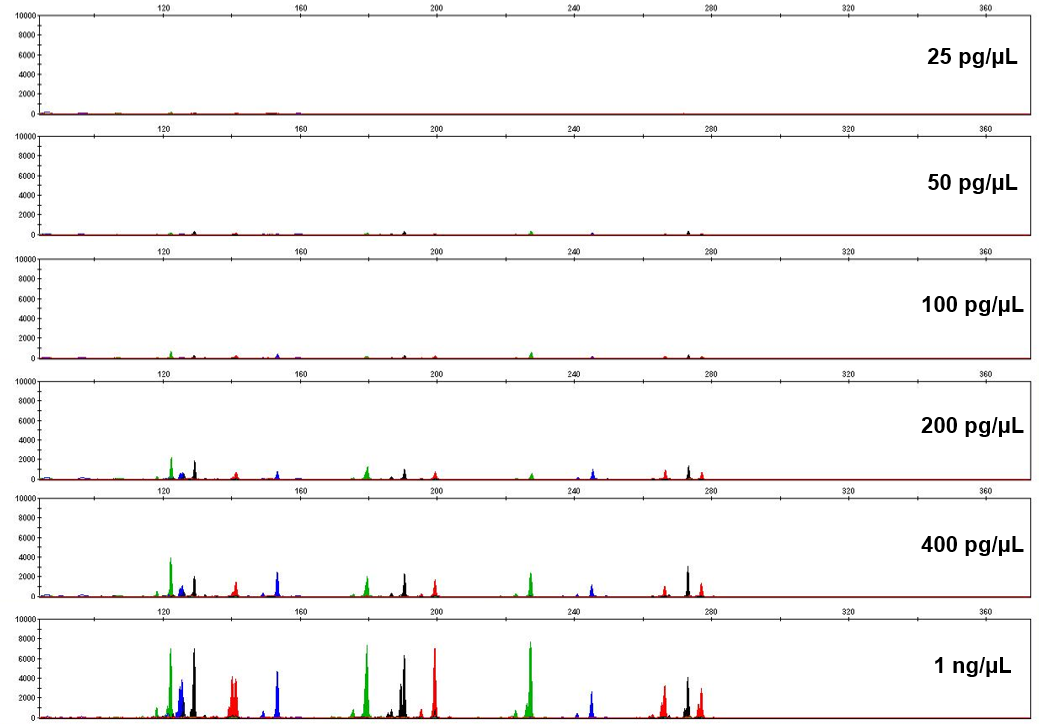

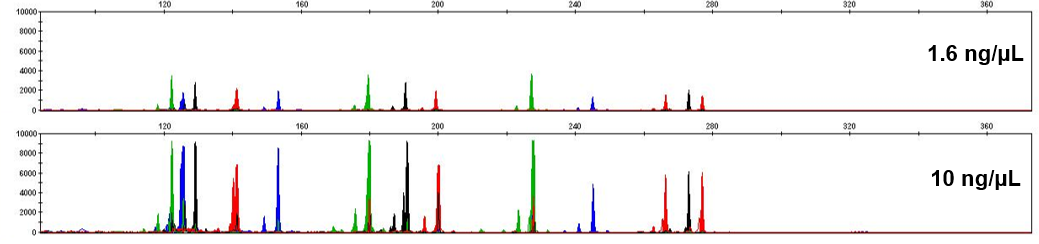


**Supplementary Figure S3.** Representative electropherograms of the sensitivity study.

**A**
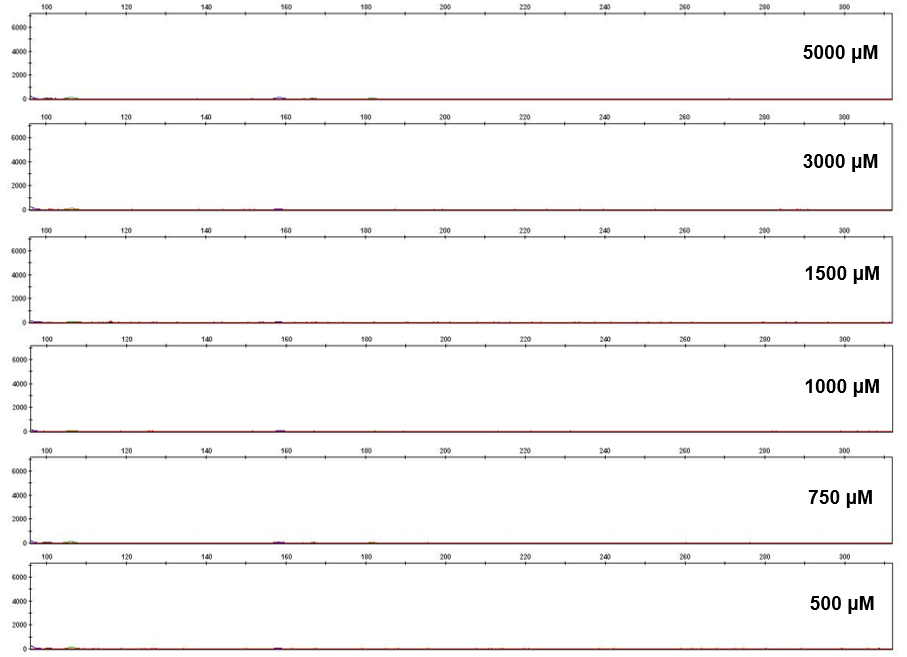

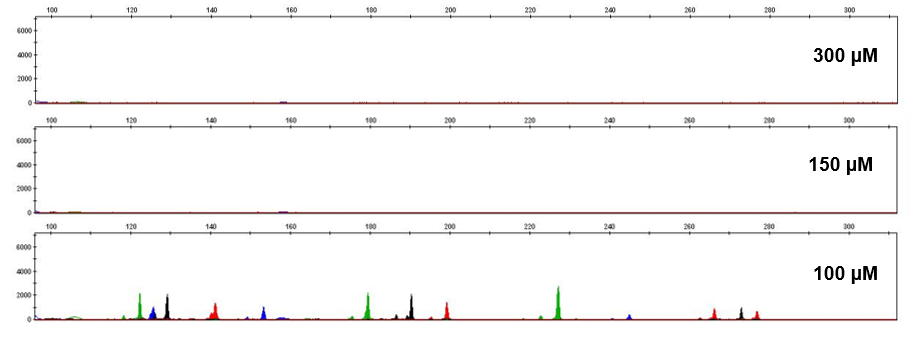


**B**
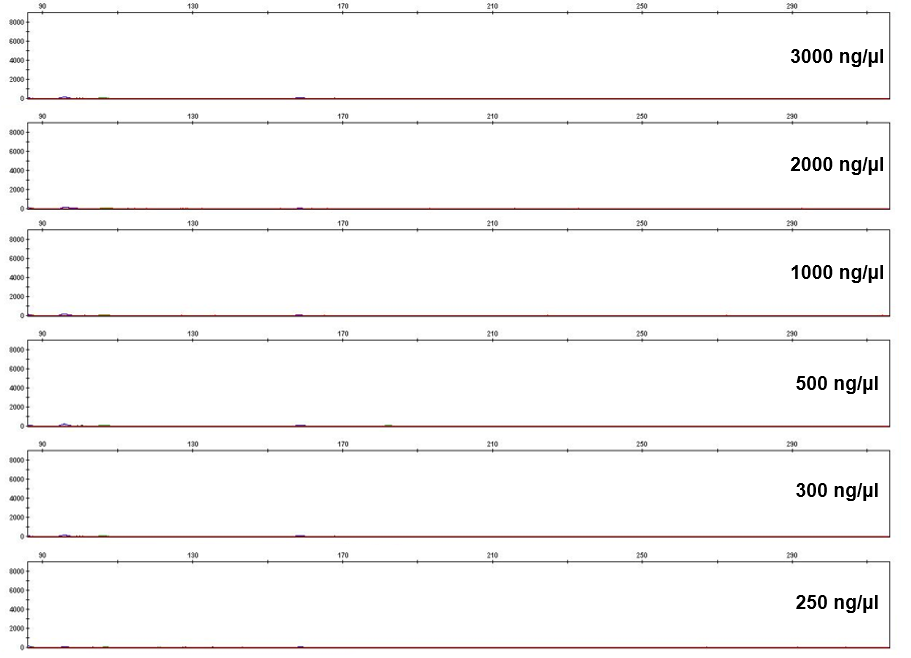

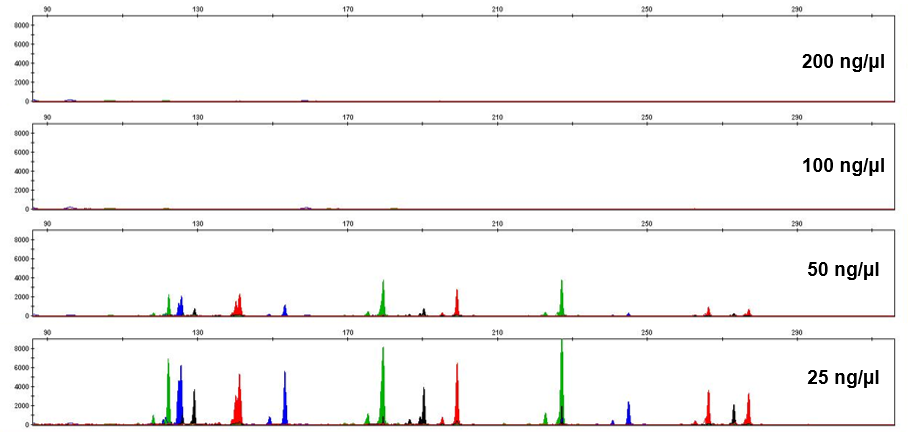


**Supplementary Figure S4**. Representative electropherograms of the effect of haematin (A) and of humic acid (B) on DNA typing.


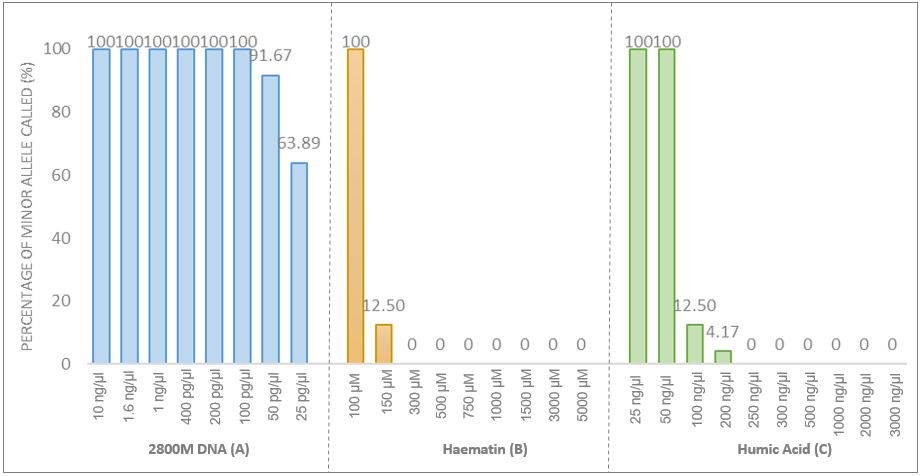


**Supplementary Figure S5.** Percentages of allele recovery for sensitivity studies through the analysis of positive control 2800M at different concentrations (A), as well as for stability studies including haematin (B) and humic acid (C) as inhibitors.


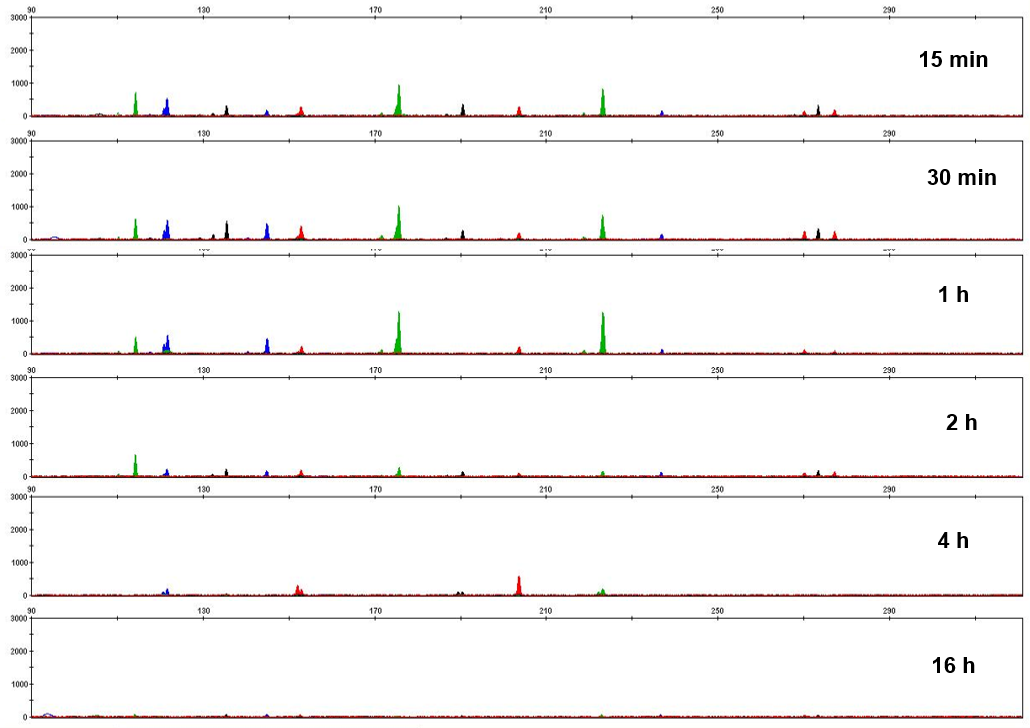


**Supplementary Figure S6.** Representative electropherograms of the artificially degradation tests performed for different times of digestion with DNase I.


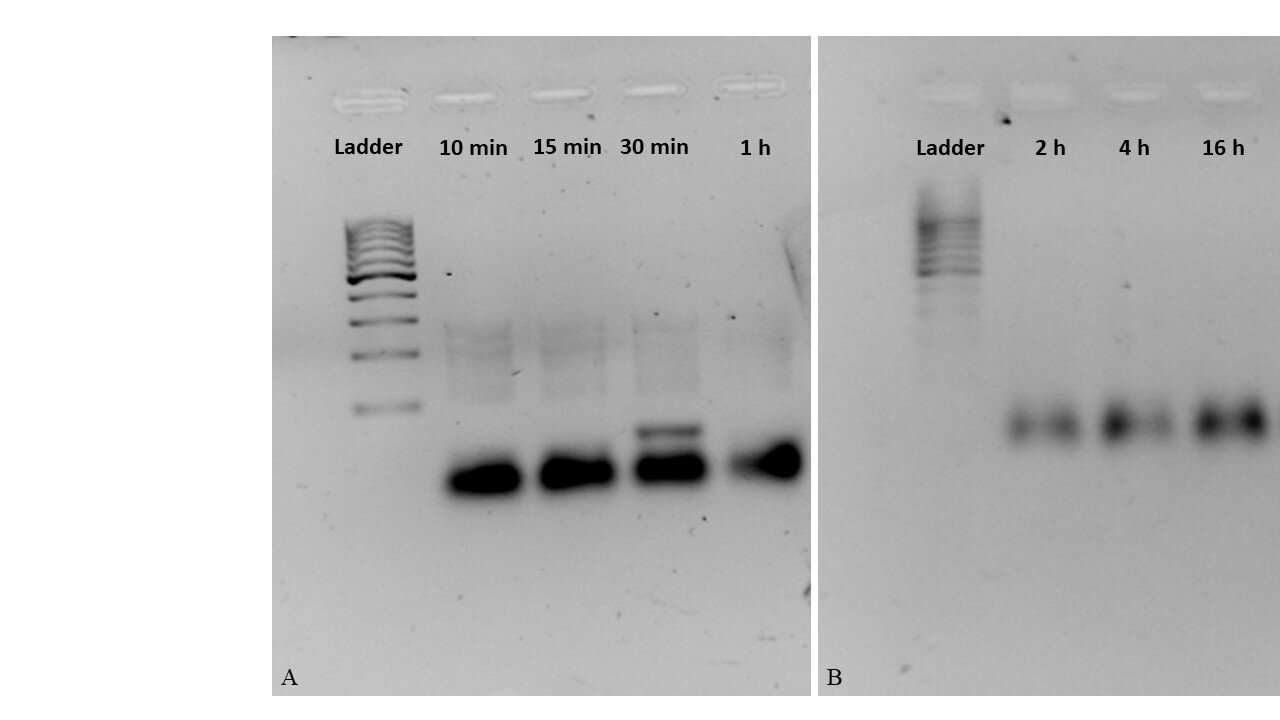


**Supplementary Figure S7.** Gel electophoresis results after different times of digestion (A: 10 min, 15 min, 30 min and 1 h; B: 2 h, 4 h and 16 h) with DNase I.


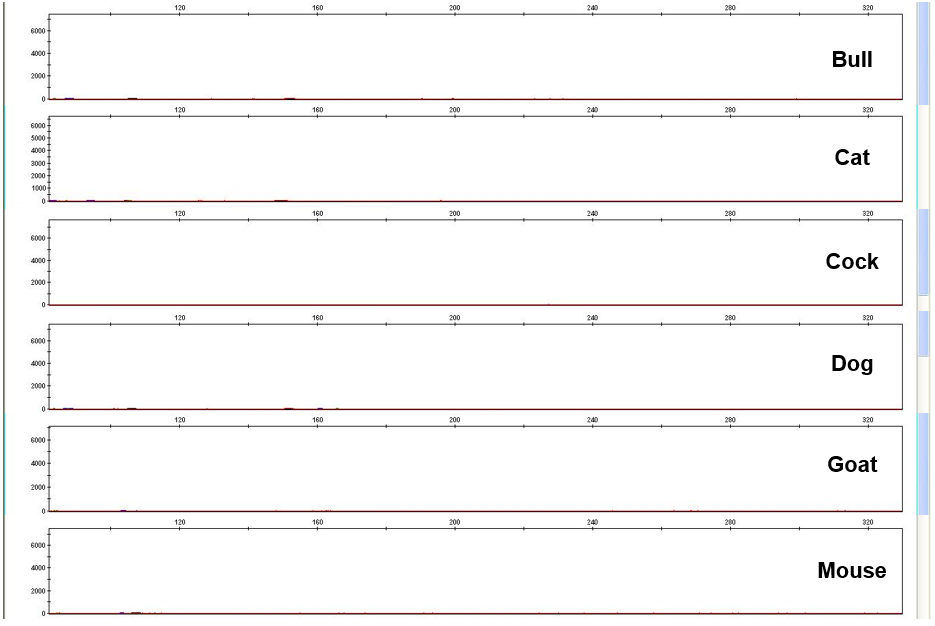

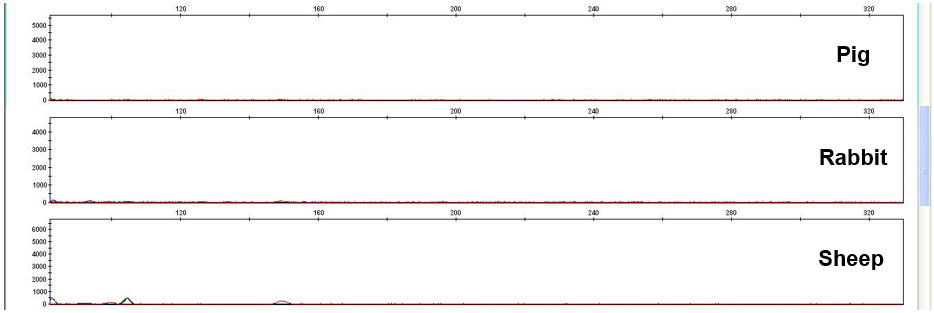


**Supplementary Figure S8.** Representative electropherograms of different animal species to test human specificity.

**A**
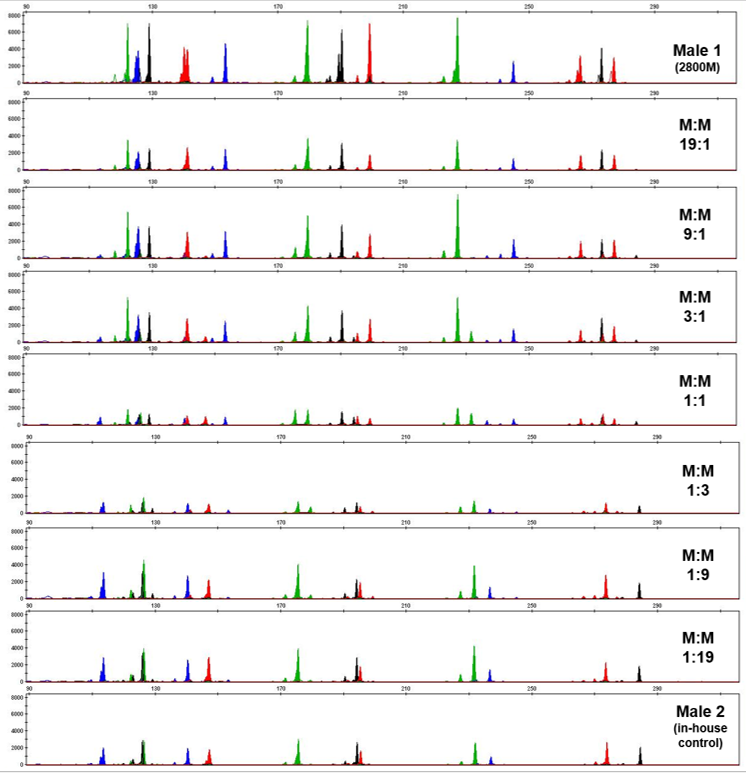


**B**
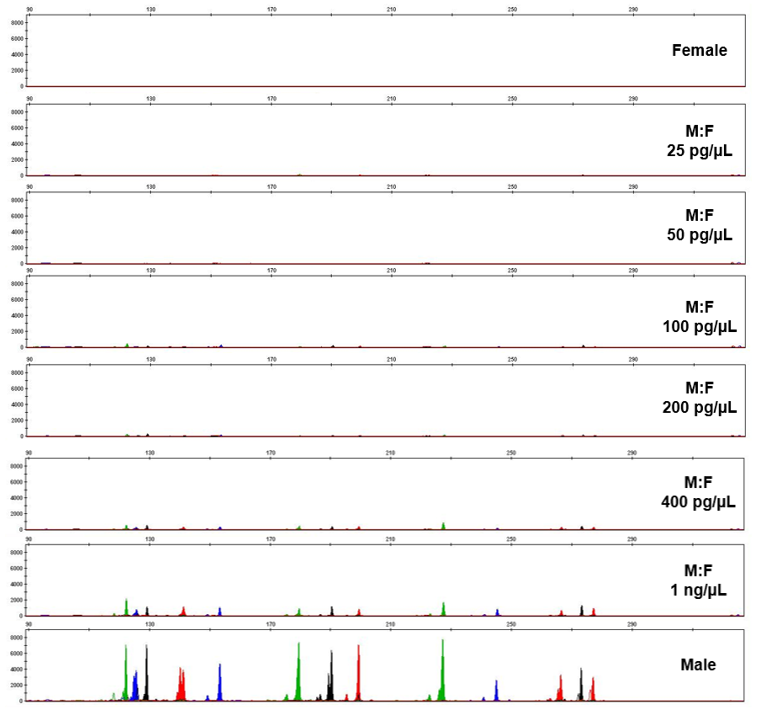


**Supplementary Figure S9**. Representative electropherograms of the mixture detection tests performed for male:male (A) and male:female (B).
